# Prevalence and Correlates of Smokeless Tobacco Consumption among Married Women in Rural Bangladesh

**DOI:** 10.1371/journal.pone.0084470

**Published:** 2014-01-08

**Authors:** Mohammad Shakhawat Hossain, Kypros Kypri, Bayzidur Rahman, Iqbal Arslan, Shahnaz Akter, Abul Hasnat Milton

**Affiliations:** 1 School of Medicine and Public Health, Faculty of Health, The University of Newcastle, New Lambton Heights, New South Wales, Australia; 2 School of Public Health and Community Medicine, The University of New South Wales, Sydney, New South Wales, Australia; 3 Bangabandhu Sheikh Mujib Medical University, Dhaka, Bangladesh; 4 Directorate General of Health Services, Dhaka, Bangladesh; The Ohio State University, United States of America

## Abstract

**Objective:**

To estimate the prevalence and identify correlates of smokeless tobacco consumption among married rural women with a history of at least one pregnancy in Madaripur, Bangladesh.

**Materials and Methods:**

We conducted a cross-sectional survey using an interviewer administered, pre-tested, semi-structured questionnaire. All women living in the study area, aged 18 years and above with at least one pregnancy in their lifetime, who were on the electoral roll and agreed to participate were included in the study. Information on socio-demographic characteristics and smokeless tobacco consumption was collected. Smokeless tobacco consumption was categorized as ‘Current’, ‘Ever but not current’ and ‘Never’. Associations between smokeless tobacco consumption and the explanatory variables were estimated using simple and multiple binary logistic regression.

**Results:**

8074 women participated (response rate 99.9%). The prevalence of ‘Current consumption’, ‘Ever consumption but not current’, and ‘Never consumption’ was 25%, 44% and 31%, respectively. The mean age at first use was 31.5 years. 87% of current consumers reported using either Shadapata or Hakimpuree Jarda. Current consumption was associated with age, level of education, religion, occupation, being an income earner, marital status, and age at first use of smokeless tobacco. After adjustment for demographic variables, current consumption was associated with being over 25 years of age, a lower level of education, being an income earner, being Muslim, and being divorced, separated or widowed.

**Conclusion:**

The prevalence of smokeless tobacco consumption is high among rural women in Bangladesh and the age of onset is considerably older than that for smoking. Smokeless tobacco consumption is likely to be producing a considerable burden of non-communicable disease in Bangladesh. Smokeless tobacco control strategies should be implemented.

## Introduction

Nearly six million people die each year as a result of tobacco use [1]_ENREF_1, accounting for 12% of global adult mortality [2]. If current tobacco use patterns continue, it will cause some 10 million deaths each year by 2020 [3]. Tobacco exposure is the single greatest preventable cause of morbidity, disability and mortality [4], [5]. Tobacco can be consumed both in smoke and smokeless form. Smokeless tobacco consumption (STC) causes oral, head and neck cancer, diabetes, hypercholesterolemia, myocardial infarction and adverse effects on pregnancy [6].

The term *smokeless tobacco* refers to more than 30 different products, broadly categorized as ‘spit tobacco’ or ‘chewing tobacco’ [6]–[9]. Tobacco is being chewed in multiple forms in south Asia: betel quid, leaf alone, leaf with lime, tobacco with areca nut preparation, and tobacco water [9], [10]. Generally, sun or air cured smokeless tobacco can be used by itself in unprocessed, processed or manufactured form [10]. Smokeless tobacco products are often made domestically in south Asia and are also widely available in markets. Usually when tobacco leaves turn yellow and brownish spots start appearing, the leaves are laid in fields for uniform drying, tied into bundles moistened with water or molasses, and stored for fermentation for a couple of weeks. The bundles are then separated and dried again, and the leaves are cut into various sizes. A variety of smokeless tobacco products have been manufactured on a large scale, commercially marketed and sold in different kinds of packets and containers [10]. The types of smokeless tobacco products in use vary by region: Snus and Snuff in Europe and North America, Gutka and Jarda in Asia, and Toombak in Africa [6], [7], [9], [11].

STC is common among south Asian people of both sexes [8], [12]_ENREF_1. More than one third of total tobacco consumption in this region is in the form of smokeless tobacco [7], [8], [13]. WHO estimates show that STC among women in south Asia is a major public health threat in Bangladesh (prevalence: 32.6%), India (18.4%), Sri Lanka (6.9%) and Nepal (6%) [14]. India is the only country in the world where overall STC is nearly twice as prevalent as smoking at 26% vs. 14% respectively, representing over 300 million users in India and nearly 26 million in Bangladesh [15], [16].

In south Asia traditional values and social norms do not favor smoking by the young or by women, but there is no such taboo against STC [6] which is integral to south Asian culture [17], being incorporated in traditional values, spirituality, beliefs, festivals, lifestyle, and rituals such as marriage and popular entertainment [6], [18]. Its perceived medicinal value for curing toothache, headache and stomach ache leads many adults to become users. Some parents even encourage their children to use smokeless tobacco [6]. Curiosity, peer pressure, and offers by friends and acquaintances contribute to initiation of use [19].

The long awaited Smoking and Tobacco Product Usage (Control) (Amendment) Act 2013 was passed recently in the parliament of Bangladesh, and is expected to reduce STC. The law includes a provision to imprison or fine those responsible for displaying tobacco advertising, makes the owners of restaurants and businesses culpable for breaches of the smoke free law, doubles the fine applied to those using tobacco in smoke free public areas, and empowers local health and government officers to I mpose penalties on law-breakers. Smokeless tobacco products, such as Jarda, Shadapata, and Gul, have now been identified as tobacco products under the law. A weakness of the previous law was that it did not include smokeless tobacco products, making it difficult to apply legal provisions to the products consumed by a majority of Bangladeshis, including powdered tobacco (Gul) and chewing tobacco (Jarda and Khainee). The Act has brought these smokeless tobacco products under the purview of the law so that regulations now apply to all smokeless tobacco products.

STC is more common among lower socioeconomic groups in Bangladesh such as poor, semi-skilled manual workers, unemployed people, and those with less education [20]. A number of studies reported that in Bangladesh, 23–34% of women in rural areas are estimated to use smokeless tobacco [10], [21]–[24]. The prevalence of at least one form of daily tobacco use (smoking or smokeless) in Bangladesh ranges between 33% and 41% [25], [26]. In 2009 the population prevalence of STC was estimated to be 27%, with similar rates in men (26%) and women (28%), and higher rates in rural areas (29%) than urban areas (23%) [16]. Some studies suggest that Bangladesh has considerably higher rates of tobacco consumption including smokeless tobacco than India and Pakistan [21].

Despite the health hazards of STC, there have been only a few studies conducted on STC in Bangladesh. The aim of this large cross-sectional study was to estimate the prevalence and correlates of STC among women living in rural areas of Bangladesh.

## Materials and Methods

### Ethical approval

We obtained ethical approval from the University of Newcastle's Human Research Ethics Committee, Australia and from the Bangladesh Medical Research Council. An information sheet describing the purpose of the study and individuals' rights as study participants was handed to the participants to read. For individuals with inadequate literacy, the information sheet was read out by the interviewers. Written informed consent was then obtained from each person. A thumb impression was obtained from those who were unable to sign the consent form.

### Study area and population

This cross-sectional survey was conducted in Bangladesh between June and September 2011. Bangladesh is divided into 64 districts in seven administrative divisions. We selected two Local Government Areas: Jhaudi and Ghotmajhee of Madaripur district which are located 220 kms south of Dhaka, the capital city. Total populations for Jhaudi and Ghotmajhi are 17,708 (9027 women; 52.3% >18 years) and 19784 (9888 women; 51.4% >18 years), respectively. The population of interest was married women aged 18 years and above with at least one pregnancy in their lifetime and who were on the electoral roll.

### Measurement of outcome variable

‘Current consumption’ was defined as consuming smokeless tobacco at least three times daily. ‘Ever consumption but not current’ was defined as having ever consumed smokeless tobacco in the respondent's lifetime and not consuming currently. ‘Never consumption’ was defined as no STC in the respondent's lifetime. We also asked respondents to indicate which smokeless tobacco products they had used.

### Data collection procedure

After obtaining informed written consent, information was collected on the prevalence of STC, knowledge and attitudes about STC, and socio-demographic information using an interviewer administered questionnaire by face-to-face interview, with measures of: age, marital status, income, employment, education, and religion. The questionnaire was developed by the investigators and was not derived from other sources. Later on, the questionnaire was finalized following pretesting at Gohinokul, a village located outside the study area but with similar population characteristics. All of the 37 interviewers were Bangladeshi women residing in the study area. They had completed at least 15 years of education and had experience in conducting interviews, surveys and using the census method. The interviewers also received a week of training on data collection techniques by the investigators and experts. One of the co-investigators (MH) supervised the fieldwork. Two percent of the completed questionnaires were cross checked randomly by this co-investigator. We conducted a door-to-door survey interviewing all the eligible women living in the study area.

### Statistical analysis

Frequency tables and summary statistics were obtained to check missing data, out-of-range values, and to assess distributions of continuous variables. Logic checks were undertaken. Categorical variables were reported as proportions. Prevalence of STC was estimated as a proportion and chi-square tests were used to compare the demographic characteristics of ‘Ever consumption but not current’, ‘Current consumption’ and ‘Never consumption’. Associations between the prevalence of STC (never vs. current consumption) and the explanatory variables were investigated using simple and multiple binary logistic regression. A backward elimination method was used to decide the final multivariable model. Any variable that was significant at the 15% level in the univariate logistic regression model was included in the base model. The final model was based on the statistical significance of the covariates. The criterion we followed was to retain all the variables significant at the 5% level in the multivariate model. We excluded variables from the base model using backward elimination based on their P-value starting with the variable with the highest P-value greater than 0.05. After forming the final model with the backward elimination method we checked for multicollinearity by estimating a variance inflation factor (VIF). A VIF of >10 was considered indicative of multicollinearity between two or more variables. A decision was made on which of the collinear variables to keep based on the context. Since VIF cannot be estimated from logistic regression we fitted a multivariate linear regression model only for the purpose of estimating VIFs [27]. Data were analysed using Stata version 12 [28].

## Results

We visited all the households (n = 7518) in the study area and identified 8082 eligible women for this study. When approached, only eight out of these eligible women refused to participate in the study yielding a participation rate of 99.9%. Only eight eligible women refused to participate. Participants' mean age was 38.5 (SD ±15.3) years with a range of 18 to 96 years. Nearly 60% of the respondents had no formal education. Most participants (95%) were Muslim, 85% were married, and 61% were housewives. The demographic characteristics of the study participants according to their STC status are presented in Table 1 which shows that 25% were current consumers, 44% had ever consumed smokeless tobacco but were not current consumers, and 31% had never consumed smokeless tobacco. 87% of current consumers reported they use either Shadapata (45%) or Hakimpuree Jarda (42%). All of the other products were less commonly used: 4% of current consumers used Gul, 3% Baba Jarda, 3% Khainee, 2% Pan Masala, and 0.7% Gutka.

**Table 1:** Smokeless tobacco consumption by demographic factors (n = 8074).

Variable	Never consumption (n = 2488)	Ever consumption but not current (n = 2027)	Current consumption (n = 3559)	*P* Value
**Overall prevalence**	30.8% (95% CI: 29.8% – 31.8%)	44.1% (95% CI: 42.9% – 45.2%)	25.1% (95% CI: 24.2% – 26.1%)	
**Age**				
≤24 years	565 (52.1%)	468 (43.2%)	54 (4.97%)	
25 to 44 years	1539 (43.1%)	1435 (40.1%)	601 (16.8%)	<0.001
≥45 years	384 (11.2%)	1656 (48.5%)	1372 (40.3%)	
**Level of education**				
No formal education	977 (24.7%)	1806 (45.6%)	1176 (29.7%)	
Primary	768 (27.7%)	1274 (45.9%)	734 (26.4%)	<0.001
Secondary	672 (56.3%)	416 (34.9%)	105 (8.80%)	
Tertiary	71 (48.6%)	63 (43.2%)	12 (8.22%)	
**Religion**				
Muslim	2330 (30.3%)	3469 (45.1%)	1894 (24.6%)	<0.001
Hindu and others	158 (41.5%)	90 (23.6%)	133 (34.9%)	
**Income earner**				
Yes	238 (40.0%)	152 (25.5%)	205 (34.5%)	<0.001
No	2250 (30.1%)	3407 (45.5%)	1822 (24.4%)	
**Occupation**				
Housewife	2264 (45.6%)	1397 (28.2%)	1299 (26.2%)	<0.001
Unemployed	188 (6.20%)	2140 (70.5%)	707 (23.3%)	
Employed	36 (45.5%)	22 (27.9%)	21 (26.6%)	
**Marital status**				
Currently Married	2338 (34.0%)	2953 (42.9%)	1592 (23.1%)	<0.001
Divorced/Widowed	150 (12.6%)	606 (50.9%)	435 (36.5%)	
**Age at first use of STC**				
≤24 years	17 (3.68%)	26 (5.62%)	419 (90.7%)	0.001
25 to 44 years	51 (5.30%)	84 (8.70%)	828 (86.0%)	
≥45 years	20 (10.1%)	23 (11.6%)	155 (78.3%)	

Overall, STC was univariately associated with age, level of education, unemployment, being an income earner, marital status, and age of first use (Table 2). When we included all of these variables in the multivariate model all were significantly associated with the outcome but there was a high collinearity between current age and age at first use (VIF  = 10.2). Accordingly, age at first use was excluded from the final model (Table 2). After multivariate adjustment, current consumption was associated only with older age, no formal education, religion, occupation, being an income earner and marital status (Table 2). The average age at first use was 31.5 (SD ±11.7) years.

**Table 2:** Unadjusted and adjusted odds of current STC (n = 4515).

Variables	Unadjusted Odds Ratio (95% CI)*	*P* Value	Adjusted Odds Ratio (95% CI)*	*P* Value
**Age**				
≤24 years	1		1	
25 to 44 years	4.08 (3.04 – 5.48)	<0.001	3.08 (2.26 – 4.20)	<0.001
≥45 years	37.3 (27.6 – 50.5)	<0.001	19.7(14.2– 27.2) P for trend <0.001	<0.001
**Level of education**				
No formal education	1		1	
Primary	0.16 (0.12 – 0.20)	0.001	0.42(0.32 – 0.55)	<0.001
Secondary	0.17 (0.09 – 0.32)	<0.001	0.39 (0.19 – 0.78)	<0.01
Tertiary	1.25 (1.10 – 1.43)	<0.001	1.16 (0.97 – 1.39) P for trend = 0.002	0.09
**Religion**				
Muslim	1		1	
Hindu and others	1.03 (0.81 – 1.31)	<0.77	0.46 (0.31 – 0.69	<0.001
**Income earner**				
Yes	1		1	
No	0.61 (0.51 – 0.73)	<0.001	2.08 (1.48 – 2.91)	<0.001
**Occupation**				
Housewife	1		1	
Unemployed	6.55 (5.50 – 7.80)	0.001	7.00(5.45 – 8.98)	<0.001
Employed	1.01 (0.59 – 1.74)	0.95	2.10 (1.02 – 4.31)	0.04
**Marital status**				
Currently Married	1		1	
Divorced/Widowed	4.25 (3.50 – 5.18)	<0.001	1.81(1.42 – 2.29)	<0.001
**Age at first use of STC**				
≤24 years	1		N/A**	
25 to 44 years	0.65 (0.37 – 1.15)	0.14		
≥45 years	0.31 (0.16 – 0.61)	<0.001		

*The odds ratio presents the odds of using smokeless tobacco among current users compared to never users.

**Excluded from the multivariate model because of high collinearity with age.

## Discussion

A quarter of rural women in Madaripur, Bangladesh are current consumers of smokeless tobacco, and another 44% have used in their lifetime. The average age at onset of STC was 31.5 years and 87% of current consumers report using either Shadapata or Hakimpuree Jarda. Current consumption is associated with older age, no formal education, occupation, being an income earner and marital status.

The main strengths of the study include the large population recruited with a census method, door-to-door contact and using a pre-tested questionnaire to conductface-to-face interviews. We invited all eligible women from the study area and collected detailed information on consumption and relevant socio-demographic variables with negligible non-response. Recruitment and training of female interviewers were undertaken so that women would feel free to participate and respond candidly.

The findings may be generalized to rural Bangladeshi married women with a history of pregnancy but not to all women in Bangladesh. There is a risk of mis-estimation of the overall rural prevalence as the study was conducted only in one out of the total 64 districts in Bangladesh. Notably, demographic characteristics of the study area were similar with respect to sex ratio, per capita income, household size, literacy rate, life expectancy, occupation, and marital status to those in other rural areas of Bangladesh, though the proportion of Hindus was lower than the national average [29]. The cross-sectional nature of the data does not allow us to assess the trends in STC over time or to make causal inferences about the associations observed. Health status of the participants and use in other family members were not measured in this study.

The high prevalence of STC reported here is consistent with other surveys of adult samples in this nation of more than 160 million people [30]. A study conducted in 16 countries on tobacco consumption (smoking and smokeless) revealed that Bangladesh has the highest prevalence of STC in the world among women (29%), more than 50% greater than in India (18%) [5]. Given that social norms governing smoking and STC appear similar across south Asia, it is unclear why Bangladesh should have a higher prevalence of STC among women. STC is firmly embedded in the traditions of south Asia and enjoyed, even revered, in several social classes. The most obvious motive for STC is social affability, in a way similar to westerners drinking coffee together. The key to its widespread patronage, though, lies in its consumption for perceived medicinal value, its use in worship as thanks to God for wellbeing, and in ceremonies including marriage and celebration of circumcision [31].

The late onset of STC found here contrasts with the early onset of smoking in many high and low income countries [32]. There may be value in investigating whether late initiation among women may reflect concerns about the negative effect of STC on appearance. Social norms regarding STC need to be better understood because economic development may modify norms and potentially remove barriers to earlier initiation. The prevalence of STC is higher among divorced and widowed women than among currently married women. Social stigma attached to smoking may have influenced their uptake of STC [31]. Earlier initiation would be expected to increase the disease burden by increasing users' duration of lifetime exposure to carcinogens.

The locally grown Shadapata and Hakimpuree Jarda are the most common forms of smokeless tobacco consumed by rural women presumably due to their low price and availability. One packet of smokeless tobacco, enough to last the typical user a week or more, costs only 8–10 taka (USD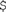 0.10), <1% of the average weekly wage in rural areas. Price is therefore not a barrier to consumption the way it is in many high income countries for cigarettes. However, the smokeless tobacco is also quite expensive in many high-income countries, not only tobacco.

The variables associated with STC in this study have also been identified in India [33]. STC and smoking are most common in the least educated population groups in India and Sri Lanka [33]. Low education is a stronger predictor of STC than household wealth for both men and women [20]. It is likely that poorly educated people are less aware of the health hazards of STC, more likely to find themselves in conditions predisposing them to initiation of STC, and more likely to have a higher degree of fatalism and overall risk taking behavior [32].

## Conclusions

Given the morbidity and mortality from STC and the high prevalence of use, regulating the production, marketing and sale of smokeless tobacco in Bangladesh should be a public health priority. In Bangladesh, tobacco smoking and STC are strongly associated with social disadvantage (i.e., low socio-economic status, less education). A comprehensive ban on tobacco advertising, promotion and sponsorship needs to be implemented according to the standard outlined in ‘Article 13’ in the WHO Framework Convention on Tobacco Control. Display and visibility of smokeless tobacco products at points of sale constitutes advertising and promotion and should therefore be banned [34]. In addition to proper enforcement of the new law, there is a need for a nationwide campaign educating people in rural areas about the law and health risks of STC.
